# Guide-Extension Catheter-Assisted Bail-Out Thrombus Aspiration During PCI for Thrombus-Rich Acute Coronary Syndromes: Contemporary Review and Clinical Case Examples

**DOI:** 10.3390/jcm15145582

**Published:** 2026-07-16

**Authors:** Josip Andelo Borovac, Mislav Lozo, Jaksa Zanchi, Anteo Bradaric, Dino Miric, Nikola Crncevic, Andrija Matetic, Mladen I. Vidovich, Mihajlo Kovacic, Claudiu Ungureanu, George Dangas

**Affiliations:** 1 Division of Ischemic Heart Disease, Department of Cardiovascular Diseases, University Hospital of Split (KBC Split), 21000 Split, Croatia; mislav.lozo@gmail.com (M.L.); zanchi.jaksa@gmail.com (J.Z.); anteo.bradaric@gmail.com (A.B.); dino.miric@gmail.com (D.M.); ncrncev1@gmail.com (N.C.); andrija.matetic@gmail.com (A.M.); 2 Department of Pathophysiology, School of Medicine, University of Split, 21000 Split, Croatia; 3 Division of Cardiology, Department of Medicine, University of Illinois at Chicago, Chicago, IL 60612, USA; miv@uic.edu; 4 Department of Interventional Cardiology, County Hospital Cakovec, 40000 Cakovec, Croatia; mihajlo1983@gmail.com; 5 Department of Cardiology, Jolimont Hospital, 7100 La Louviere, Belgium; ungureanu@cardioact.com; 6 The Zena and Michael A. Wiener Cardiovascular Institute, Mount Sinai Fuster Heart Hospital, Icahn School of Medicine at Mount Sinai, New York City, NY 10029, USA; george.dangas@mountsinai.org

**Keywords:** acute coronary syndrome, coronary thrombosis, guide-extension catheter, mechanical thrombectomy, myocardial infarction, no-reflow, percutaneous coronary intervention, slow-flow, thrombus aspiration

## Abstract

Large intracoronary thrombus burden during percutaneous coronary intervention (PCI) for acute coronary syndrome (ACS) remains technically challenging when conventional manual aspiration is ineffective or dedicated thrombectomy systems are unavailable, unsuitable, or undeliverable. Routine aspiration thrombectomy is not guideline-supported, but selective bail-out thrombus-removal strategies remain relevant in refractory thrombus-rich PCI. This contemporary narrative review, supplemented by six retrospective single-center clinical examples, describes off-label guide-extension catheter (GEC)-assisted thrombus aspiration and places it within the current thrombectomy landscape, including manual aspiration catheters, sustained mechanical aspiration platforms, and stent-retriever-based systems. The heterogeneous examples, predominantly involving the right coronary artery, are educational and hypothesis-generating only: they illustrate procedural mechanics, patient selection, technical pitfalls, and risk mitigation, but do not provide efficacy or safety evidence. GEC-assisted aspiration may be considered when bulky proximal thrombus cannot be captured by smaller catheters or when a GEC can be positioned coaxially near the thrombus face under uninterrupted negative pressure. The technique remains operator-dependent, off-label, and anatomically constrained and should be regarded as a contingency maneuver rather than an alternative to purpose-built systems with more formal device-specific evaluation. Prospective registries and comparative studies are required before efficacy, safety, or relative value can be established.

## 1. Introduction

Large intracoronary thrombus burden during acute coronary syndrome (ACS) increases the risk of distal embolization, microvascular obstruction, no-reflow or slow-flow, larger infarct size, impaired myocardial recovery, and adverse clinical outcomes [1,2,3,4]. Early enthusiasm for manual aspiration was driven by studies such as TAPAS and EXPIRA, in which aspiration with the Export catheter or similar manual systems improved surrogate markers of reperfusion and infarct size [5,6]. However, larger randomized trials subsequently failed to confirm a hard-outcome benefit of routine aspiration and raised concern regarding stroke, particularly in the TOTAL trial [7,8,9]. The 2023 ESC guidelines do not recommend routine thrombus aspiration but allow that selective aspiration may be considered when a large residual thrombus remains after vessel opening with a guidewire or balloon; similarly, the 2025 ACC/AHA/ACEP/NAEMSP/SCAI guideline assigns routine manual aspiration during primary PCI for STEMI a Class 3 (no benefit) recommendation [10,11].

Despite negative randomized evidence for routine manual aspiration, thrombus-rich ACS remains a daily procedural problem. Selective thrombus removal may still be necessary when conventional balloon angioplasty or direct stenting cannot be performed safely in a large thrombotic mass, when distal embolization or no-reflow occurs, or when thrombus persists despite antithrombotic therapy. Contemporary options include conventional manual aspiration catheters, sustained mechanical aspiration systems such as the Indigo CAT RX platform, and stent-retriever-based coronary thrombectomy technologies such as NeVa and enVast [12,13,14,15,16].

Guide-extension catheters (GECs), including GuideLiner, Guidezilla, Telescope, LiquID, and similar devices, are widely used to improve guide-catheter support, coaxiality, and device delivery during complex PCI. Because of their relatively large inner lumen and deliverability into proximal or mid-coronary segments, GECs may also permit retrieval of bulky thrombus in selected bail-out scenarios. A key early feasibility experience was reported by Farooq et al., who applied a combined “forward” and “back” aspiration strategy in 30 consecutive STEMI procedures, including bail-out GuideLiner-assisted aspiration in nine patients after conventional aspiration was inadequate [17]. This off-label application has otherwise been described mainly in small series and case reports and remains unsupported by prospective comparative data [18,19,20,21,22,23,24,25].

This topic matters now because the procedural landscape has evolved while real-world device availability remains variable. Dedicated aspiration and thrombectomy technologies are increasingly described and, in some cases, formally evaluated, but many catheterization laboratories may not have immediate access to these platforms in time-critical ACS procedures. A practical review of GEC-assisted aspiration as an off-label contingency technique may therefore be useful for operators facing refractory thrombus during percutaneous coronary intervention (PCI), provided that the technique is framed conservatively and not presented as a validated alternative to dedicated thrombectomy systems.

Because no dedicated consensus document specifically addresses GEC-assisted coronary thrombus aspiration, procedural decisions should be individualized and grounded in contemporary ACS guidance, device availability, thrombus morphology, coronary anatomy, and operator experience. Accordingly, this review should not be interpreted as a guideline or pre-guideline document.

## 2. Literature Search Strategy

This manuscript is a contemporary narrative review with practical clinical examples; it is not a systematic review. PubMed/MEDLINE and Google Scholar were searched from inception through May 2026 using combinations of “guide extension catheter”, “mother-and-child”, “Guidezilla”, “GuideLiner”, “Telescope”, “LiquID”, “Export”, “manual aspiration catheter”, “thrombus aspiration”, “thrombectomy”, “no-reflow”, “slow-flow”, “CAT RX”, “CHEETAH”, “NeVa”, “enVast”, “stent retriever”, “continuous aspiration”, “forward aspiration”, and “back aspiration”. Guideline statements, randomized thrombectomy trials, device-specific studies, nonrandomized feasibility studies, small series, and case reports were screened. No review protocol, duplicate screening, or formal risk-of-bias assessment was used. The evidence base for GEC-assisted aspiration remains limited to nonrandomized feasibility data, small series, and case reports.

## 3. Clinical Case Examples

Supplementary File S1 presents six clinical examples from real-world practice. The examples were selected retrospectively from thrombus-rich PCI procedures in which GEC-assisted aspiration was used after conventional thrombus-management strategies were inadequate, unavailable, unsuitable, or technically unsuccessful. Clinical, angiographic, procedural, and immediate in-hospital outcomes were reconstructed from procedural records. Thrombus burden, flow restoration, no-reflow or slow-flow, distal embolization, and immediate complications were assessed by operator review. No independent angiographic core laboratory, systematic neurologic assessment, or prespecified follow-up protocol was used.

The clinical examples are intentionally educational and hypothesis-generating rather than representative. All patients were male, five of six procedures involved the right coronary artery, and the clinical contexts were heterogeneous. Cases 1–4 most closely reflect thrombus-rich ACS bail-out scenarios, whereas Case 5 (subacute stent-edge thrombus in unstable angina) and Case 6 (post-stenting thrombus shift) are retained only as adjacent technical examples of thrombus retrieval. Table 1A–C summarizes baseline angiography, procedural details, and immediate outcomes. Because of the small sample size, absence of a control group, retrospective selection, and operator adjudication, no inference can be made regarding procedural success rates, stroke risk, mortality, or comparative performance against dedicated aspiration or thrombectomy systems. The absence of a reported immediate complication is descriptive only and should not be interpreted as a safety claim.

## 4. Patient Selection: Potential Bail-Out Scenarios and Situations to Avoid

**Figure 1:**
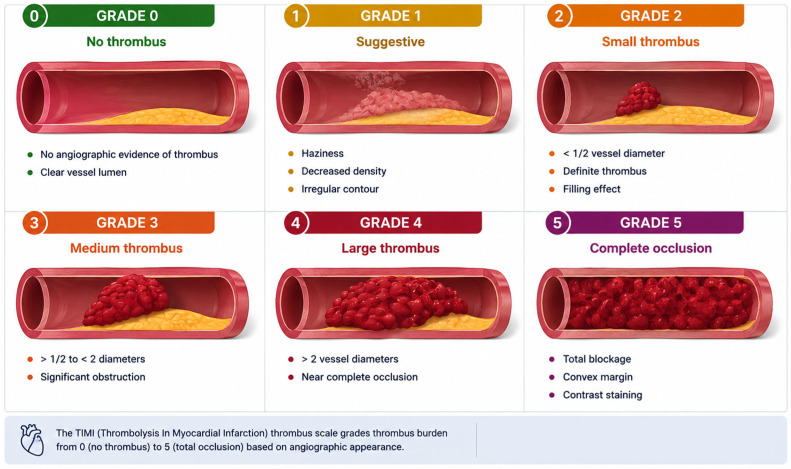
TIMI thrombus scale angiographic classification of coronary thrombus burden. Adapted from Gibson, C.M.; de Lemos, J.A.; Murphy, S.A.; Marble, S.J.; McCabe, C.H.; Cannon, C.P.; Antman, E.M.; Braunwald, E.; TIMI Study Group. Combination therapy with abciximab reduces angiographically evident thrombus in acute myocardial infarction: a TIMI 14 substudy. *Circulation* **2001**, *10*, 2550–2554 [26].

GEC-assisted aspiration may be considered only as a selective bail-out option in thrombus-rich ACS PCI. Case-informed scenarios include: (1) persistent TIMI thrombus grade 4–5 with impaired epicardial flow after wire crossing, limited lesion opening, and appropriate antithrombotic therapy; (2) failure, inadequacy, unavailability, unsuitability, or undeliverability of a dedicated aspiration or mechanical thrombectomy system; (3) a bulky, discrete proximal thrombus that can be approached coaxially without unsafe deep seating; or (4) thrombus visibly corking a catheter tip and not retrievable by conventional means. Preconditions should include therapeutic anticoagulation, stable wire position, acceptable hemodynamics, a coaxial guide system without pressure damping, and a plan for closed-system withdrawal. The maneuver should be stopped if resistance, pressure damping, loss of suction, worsening flow, or loss of coaxial alignment occurs (Figure 1).

This case-informed framework is a descriptive single-center workflow and should not be interpreted as a guideline recommendation or validated clinical algorithm.

GEC-assisted aspiration should generally be avoided or abandoned in vessels < 2.5 mm, severe ostial disease, long diffusely diseased segments, marked tortuosity, pressure damping, poor guide coaxiality, inability to maintain therapeutic anticoagulation, or any situation in which deep intubation appears likely to cause vessel injury. Additional caution is warranted in cardiogenic shock or severe left ventricular dysfunction, in which prolonged manipulation, transient ischemia, pressure damping, or distal embolization may be poorly tolerated by the patient. When a dedicated thrombectomy technology with supportive clinical evaluation is available, deliverable, and anatomically suitable, it should generally be preferred over off-label GEC-assisted aspiration.

Applicability should not be inferred equally across coronary territories. The present experience was dominated by relatively large RCA anatomy, in which proximal or mid-vessel caliber and a favorable catheter course may facilitate coaxial GEC delivery. In the LAD or left main, distal embolization can jeopardize a larger myocardial territory and multiple side branches; in the circumflex artery, an acute take-off, tortuosity, and smaller caliber may limit deliverability. At bifurcations, catheter advancement or aspiration may displace thrombus into a side branch. In ectatic vessels, a GEC may be deliverable, but diffuse thrombus volume and embolic potential may exceed the capacity of a single aspiration maneuver. The all-male case set also precludes sex-specific inference; vessel size, lesion location, and catheter course, rather than sex itself, should drive technical selection.

## 5. Contemporary Thrombectomy Device Landscape

Conventional manual aspiration catheters remain the historical comparator. The Export catheter was prominently studied in TAPAS and EXPIRA, which supported feasibility and improved reperfusion surrogates, but these single-center and mechanistic data were subsequently outweighed by large randomized evidence showing no routine clinical benefit and a significantly increased stroke signal with systematic aspiration [5,6,7,8,9]. Therefore, failure of an Export-type catheter in an individual case does not establish the superiority of a GEC. Rather, it identifies a practical problem in which alternative thrombus-management strategies may be considered.

Sustained and continuous mechanical aspiration represents a more contemporary approach. The Indigo CAT RX system is a purpose-built aspiration platform designed for continuous mechanical aspiration of coronary thrombus. In A Prospective, Multicenter Study to Evaluate the Safety and Performance of the CAT RX Aspiration Catheter in Patients With a High Thrombus Burden Acute Coronary Vessel Occlusion (CHEETAH), sustained aspiration performed before PCI was associated with high rates of final TIMI 3 flow, myocardial blush grade 3, and successful thrombus removal, with no device-related serious adverse events reported [12]. Although CHEETAH was not a randomized comparison against PCI alone or against GEC-assisted aspiration, it provided a stronger device-specific clinical evidence base than case-level GEC experience.

Stent-retriever-based coronary thrombectomy is another emerging strategy adapted from neurovascular and neuroradiology thrombectomy principles. In a prospective first-in-human EuroIntervention experience, the NeVa mechanical thrombectomy device was used in ACS patients with large thrombus burden, providing structured device-specific feasibility and safety data [13]. EnVast has also been reported as a bail-out mechanical thrombectomy system after conventional thrombus aspiration failure in STEMI with massive thrombotic burden [14]. More recently, EuroIntervention published a 2026 case report describing stent retriever-assisted coronary thrombectomy with continuous aspiration, further supporting the concept that combined retrieval and aspiration may be technically feasible in selected refractory coronary thrombus scenarios [15].

Taken together, device selection should be individualized and should reflect thrombus morphology, coronary anatomy, deliverability, local availability, and operator experience. Manual thrombus aspiration catheters are familiar and simple but have limited support for routine PCI use. CAT RX provides sustained aspiration with prospective multicenter data. Stent-retriever-based systems such as NeVa and enVast are specifically engineered for clot engagement and retrieval and are being evaluated more systematically. Where dedicated thrombectomy systems are available, deliverable, and appropriate for the anatomy, they should be considered within the operator’s thrombus-management strategy because they have more device-specific evaluation than off-label GEC-assisted aspiration. However, no randomized comparison exists between these technologies and GEC-assisted aspiration. Based on our limited clinical experience, GEC-assisted thrombus aspiration may remain useful when dedicated thrombus removal devices fail, are unavailable, cannot be advanced, or when a GEC is already positioned near a large proximal thrombus and can be used without unsafe deep intubation. However, its evidence base for this indication is limited and should be acknowledged as such.

The mechanistic rationale for GEC-assisted aspiration is based on three features: a larger effective aspiration lumen compared with many conventional manual aspiration catheters, improved coaxial engagement of proximal thrombus, and the ability to retrieve thrombus en bloc when thrombus is lodged at the catheter tip. These features may be particularly relevant for bulky proximal thrombus, organized thrombus, or thrombus with structural features that limit capture by smaller aspiration catheters [27,28]. Conversely, these same features increase the importance of careful vessel selection, avoidance of forceful deep intubation, and uninterrupted negative pressure during withdrawal.

## 6. Technique Description for Selective GEC-Assisted Thrombus Aspiration

**Figure 2:**
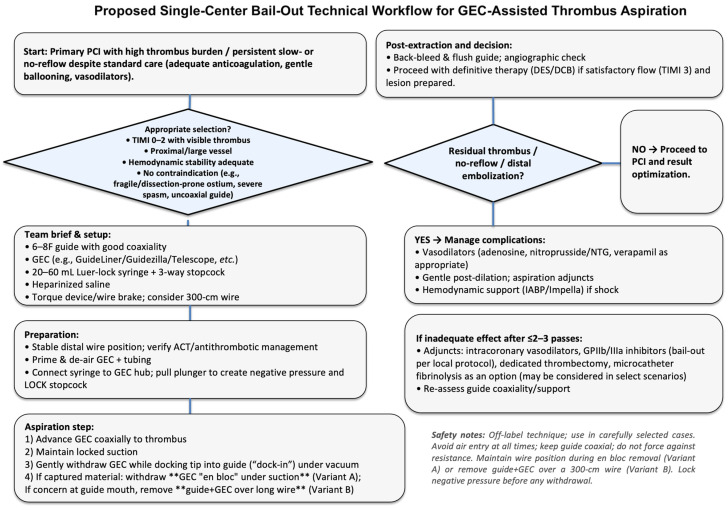
Proposed single-center bail-out technical workflow for selective guide-extension catheter-assisted thrombus aspiration. The figure is a descriptive local workflow and not a validated clinical management algorithm. ** important procedural distinction.

The following steps summarize technical considerations from a single-center experience rather than a validated procedural algorithm (Figure 2).

1. Initial preparation: Cross the lesion with a suitable coronary wire and optimize guide-catheter coaxiality. Consider microcatheter support when wiring is difficult. Deep intubation should not be attempted if the guide is non-coaxial, if pressure damping occurs, or if there is significant ostial disease or severe tortuosity.
2. GEC positioning: Advance the GEC gently over the wire to a position just proximal to the thrombus. Balloon anchoring or inchworm techniques may be considered only when necessary and only if they can be performed without excessive vessel trauma. Resistance should prompt reassessment rather than forceful advancement. Device-specific knowledge is required while relevant specifications of commonly used GEC devices are summarized in Table 2.
3. Establish uninterrupted suction and negative pressure: The entire aspiration system must be fully primed and meticulously de-aired. A 20- to 60-mL Luer-lock/VacLok syringe or equivalent suction source can be connected through a secure stopcock on the Y-connector side port. Negative pressure should be established before thrombus engagement and must remain uninterrupted while the GEC tip is in contact with thrombus. The stopcock should remain closed when components are disconnected, and contrast should not be injected through a system that may contain air or thrombotic material.
4. Thrombus retrieval: Short, controlled forward/back movements may help engage thrombus at the GEC tip; forceful advancement should be avoided. If the tip becomes corked, the GEC should be withdrawn slowly under continuous negative pressure and, when necessary, removed together with the guide as a closed unit. Before any further angiography or device delivery, the guide should be meticulously aspirated and back-bled, and the withdrawn system should be inspected and flushed outside the body. Repeated ineffective attempts, resistance, pressure damping, or worsening flow should prompt abandonment of the maneuver rather than escalation of force.
5. Adjunctive measures: Intracoronary vasodilators or other pharmacologic agents may be used for slow-flow/no-reflow according to the clinical scenario, local protocol, and bleeding risk. GP IIb/IIIa inhibitors may be considered for bail-out thrombotic complications in accordance with guideline-supported rescue use [10,11]. Selected pharmacologic options, example dosing ranges, and major adverse effects are summarized in Table 3; these values are not treatment recommendations.

Intracoronary imaging with IVUS or OCT may be considered after safe restoration of antegrade flow and reduction in thrombus volume. Imaging can help distinguish residual thrombus from plaque rupture or erosion, characterize the underlying lesion, identify stent underexpansion or malapposition, detect edge dissection or tissue prolapse, and clarify thrombus shift after stenting. OCT provides high-resolution thrombus and surface-morphology assessment but requires adequate blood clearance and additional contrast. IVUS may be preferable in large vessels, ostial disease, renal impairment, or when blood clearance is suboptimal. Imaging should not be advanced through unstable bulky thrombus or used in a way that delays treatment of ongoing ischemia, and evidence supporting routine imaging specifically after GEC-assisted aspiration is lacking.

## 7. Distal Embolization Protection, “Balloon-Block” Techniques, and Local Intraprocedural Pharmacology

Distal protection filters, distal balloon-block techniques, and combined aspiration/pharmacologic approaches should be regarded as occasional case-based adjuncts, not routine components of a structured pathway. They may be technically rational in selected large-vessel, bifurcation, or ectatic anatomy, but they can also cause branch ischemia, vessel injury, device entrapment, or thrombus displacement. Current guidelines do not specifically endorse these maneuvers for GEC-assisted aspiration [29,30].

Farooq et al. prospectively evaluated a combined “forward” and “back” aspiration strategy in 30 consecutive STEMI procedures. Forward aspiration used a conventional aspiration catheter, with bail-out GuideLiner aspiration—with or without a distally inflated balloon—when thrombus persisted; back aspiration applied negative pressure through a GuideLiner or deeply intubated guide catheter during balloon deflation and stent optimization. GuideLiner-assisted bail-out aspiration was used in 9/30 cases and back aspiration in all 30. Although angiographic flow and blush were generally maintained, distal embolization to an unprotected branch and longitudinal stent deformation were reported. These findings support technical feasibility but not routine efficacy or safety [17].

Randomized and mechanistic studies of intracoronary low-dose fibrinolysis during STEMI have not provided a consistent clinical benefit, while full-dose facilitated PCI with systemic fibrinolysis is a distinct and harmful strategy that should not be conflated with very-low-dose local intracoronary administration [31,32,33,34,35,36]. If low-dose intracoronary fibrinolytic therapy is contemplated by the operator, it should be regarded as off-label, highly selected, and subordinate to bleeding risk and institutional practice rather than presented as a recommendation [37,38,39,40].

## 8. Safety Considerations

Potential complications of GEC-assisted aspiration include vessel dissection, pressure damping and ischemia during deep seating, distal embolization/no-reflow, systemic embolization if thrombus is released during withdrawal, guide or GEC thrombosis, air embolism, and device-delivery complications such as stent stripping or longitudinal stent deformation at the GEC collar [41,42]. The stroke signal observed with routine manual aspiration in TOTAL cannot be directly extrapolated to selective GEC use, but it heightens the need for continuous negative pressure, closed-system withdrawal, meticulous guide aspiration/back-bleeding, complete de-airing before every pass, avoidance of contrast injection through a potentially contaminated system, avoidance of forceful deep intubation, and periprocedural neurologic surveillance [8,9].

The six illustrative clinical examples are inadequate to estimate the incidence of stroke or other infrequent complications. With zero reported events in six patients, the confidence interval around any rare-event rate remains too wide to support reassurance. The absence of reported complications is therefore descriptive only. Potential pitfalls and preventive or management measures are summarized in Table 4.

## 9. Discussion

Based on available evidence and the present single-center experience, GEC-assisted thrombus aspiration should be positioned as a selective, narrowly indicated bail-out maneuver rather than a review-derived clinical algorithm. Its potential value lies in coaxial engagement of bulky thrombus and a lumen that may be larger than that of some conventional manual aspiration catheters. However, the supporting evidence comprises one prospective nonrandomized feasibility study, small series, and case reports; the six examples in this review are educational and hypothesis-generating only and cannot establish efficacy, safety, or generalizability [17,18,19,20,21,22,23,24,25].

### 9.1. Alignment with Contemporary Guidelines

The contemporary ESC and ACC/AHA positions converge on avoiding routine manual aspiration while differing in how explicitly they describe selective use. The 2023 ESC ACS guidelines state that routine thrombus aspiration is not recommended but that aspiration may be considered when a large residual thrombus remains after the vessel has been opened with a guidewire or balloon [10].

The 2025 North American (ACC/AHA/ACEP/NAEMSP/SCAI) ACS guidelines classify routine manual aspiration in STEMI undergoing primary PCI as a Class 3 indication (no benefit) [11]. Neither guideline validates off-label GEC-assisted thrombus aspiration and such a method is not explicity discussed within documents (Table 5). The present technique therefore occupies, at most, a narrow therapeutic window within individualized thrombus management and should not be presented as guideline-supported.

### 9.2. Case-Informed Practical Indications and Conditions of Use

Across the six clinical examples, enabling conditions included a discrete or bulky thrombus in a vessel large enough to accommodate a 6F GEC, stable wire access, the ability to maintain uninterrupted suction, and an operator-defined reason not to continue conventional aspiration alone. Cases 1–3 used a GEC early in large RCA anatomy and therefore reflect local practice and device availability rather than evidence for routine upfront use. Case 4 most closely illustrates a final bail-out sequence after three unsuccessful Export aspiration runs and highly selected local pharmacologic escalation. Cases 5 and 6 are adjacent technical examples rather than core indications. In practice, operators should use the fewest controlled attempts necessary, reassess after each attempt, and switch or stop when the thrombus is not captured, the GEC cannot be positioned safely, or any safety threshold is crossed.

A central practical message is that GEC use must be interpreted within, not isolated from, the evolving thrombectomy landscape. Export-type manual aspiration catheters are historically important but insufficient as the only comparator. CAT RX continuous mechanical aspiration has prospective multicenter evidence in high-thrombus-burden ACS, while NeVa/enVast and related stent-retriever plus aspiration strategies are purpose-built technologies with emerging EuroIntervention and JACC Cardiovascular Interventions experience and ongoing randomized evaluation [5,6,12,13,14,15,16].

Where dedicated thrombectomy systems are available, deliverable, and appropriate for the anatomy, they should be considered within the operator’s thrombus-management strategy because they have more device-specific evaluation than off-label GEC-assisted aspiration. However, no randomized comparison exists between these technologies and GEC-assisted aspiration. Conversely, GEC-assisted aspiration may remain relevant when dedicated devices fail, are unavailable, cannot be advanced, or when a GEC is already positioned near a large proximal thrombus and can be used without unsafe deep intubation. This should be viewed as a contingency pathway rather than a competing device strategy.

The clinical examples illustrate technical feasibility in selected thrombus-rich procedures, particularly in large RCA anatomy, but they do not establish efficacy, safety, or generalizability to the LAD, circumflex, left main, bifurcation, ectatic, small-vessel, cardiogenic-shock, or severe left-ventricular-dysfunction settings. Distal embolization in the LAD or left main may jeopardize a larger myocardial territory and multiple side branches, whereas acute circumflex angulation and smaller caliber may limit GEC delivery. Bifurcations create a risk of thrombus displacement into a branch, and ectatic vessels may contain a thrombus volume that exceeds the capacity of a single aspiration maneuver. Case 5 represents subacute stent-edge thrombus in unstable angina, and Case 6 represents post-stenting thrombus shift; neither should be used to justify a broad indication or management algorithm.

**Figure 3:**
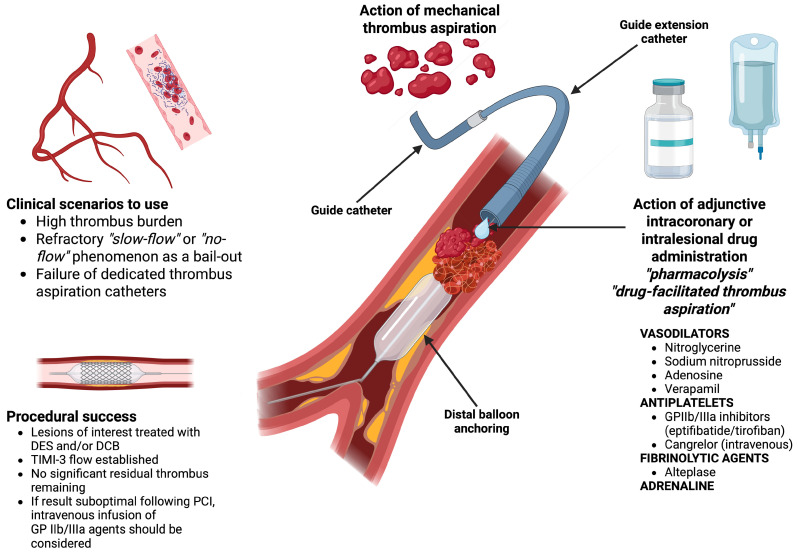
Graphical summary of potential selective bail-out scenarios, mechanical considerations, case-based adjuncts, safety limits, and procedural endpoints for GEC-assisted aspiration.

Future work should prioritize prospective registries that capture coronary anatomy, thrombus grade, device availability, aspiration approach, number of controlled attempts, adjunctive pharmacology, intracoronary imaging, neurologic outcomes, distal embolization/no-reflow, and comparative performance against modern dedicated systems. Whether GEC-assisted aspiration has any role before conventional or purpose-built thrombus-removal strategies remains an open research question; current evidence does not support routine frontline use. Until clinical outcome data are available, the approach should remain selective, protocolized, explicitly bail-out oriented, and subject to conservative stopping rules. Potential mechanical and pharmacologic adjuncts are summarized in Figure 3.

## 10. Limitations

There are notable limitations to this work. It is a narrative review supported by retrospective, single-center clinical examples; the case volume is small, uncontrolled, and vulnerable to selection, information, and publication bias. The literature search was not systematic and used no protocol, duplicate screening, or formal risk-of-bias assessment. All patients were male, five of six procedures involved the RCA, access-site information and exact aspiration-pass counts were unavailable in several records, and two cases were adjacent technical examples rather than representative primary-PCI bail-out cases. Outcomes were operator-assessed, follow-up was limited, and no systematic neurologic assessment or independent angiographic core-laboratory review was performed. The manuscript should therefore be interpreted as a technical narrative review with educational, hypothesis-generating illustrations, not as evidence of efficacy or safety.

## 11. Conclusions

GEC-assisted thrombus aspiration may be technically feasible as a selective bail-out maneuver in thrombus-rich ACS when conventional measures fail or a dedicated thrombectomy system is unavailable, unsuitable, undeliverable, or unsuccessful. It should not be used routinely or represented as a guideline-supported or validated thrombectomy strategy. The six clinical examples are educational and hypothesis-generating only. Any use requires careful anatomic selection, therapeutic anticoagulation, meticulous de-airing, uninterrupted negative pressure, closed-system withdrawal, guide aspiration/back-bleeding, and immediate abandonment for resistance, pressure damping, loss of suction, or worsening flow. Larger prospective datasets are required before efficacy, safety, or comparative value can be assessed.

## Figures and Tables

**Table 1:** Clinical, angiographic, procedural, and immediate in-hospital characteristics of the six illustrative GEC-assisted thrombus-aspiration cases. (A) Baseline and angiographic characteristics. (B) Procedural details. (C) Immediate angiographic and in-hospital outcomes.

(A)						
**Case**	**Age/Sex**	**Presentation**	**Culprit Anatomy/Lesion**	**Baseline TIMI Flow**	**TIMI Thrombus Grade**	**Access**
**1**	48/M	Subacute inferoposterior STEMI (~12 h symptom-to-door)	Proximal dominant RCA; total thrombotic occlusion	0	5	Radial
**2**	48/M	Inferior STEMI	Proximal hyperdominant RCA; total thrombotic occlusion	0	5	Radial
**3**	47/M	Inferior STEMI	Mid RCA; large mobile thrombus causing ~80% luminal obstruction	Antegrade flow present; formal grade NR	4	Radial
**4**	30/M	Inferolateral STEMI	Mid RCA/crux; thrombus extending into PD and PL branches	0	5	Radial
**5**	55/M	Unstable angina 7 days after proximal LAD PCI	Proximal LAD stent-edge intraluminal white thrombus	Patent vessel; formal grade NR	NR (large thrombus)	Radial
**6**	73/M	Inferolateral STEMI	High proximal hyperdominant RCA; total thrombotic occlusion	0	5	Radial
(B)						
**Case**	**Prior Strategy/Procedural Indication**		**GEC and Technical Approach**	**GEC Aspiration** **Attempts**	**Adjunct Pharmacology**	**Intracoronary Imaging**
**1**	Bulky proximal RCA thrombus; primary GEC use after wire crossing		6F Guidezilla II; distal 2.5 × 15-mm balloon-block; en-face engagement; closed-system removal	One aspiration sequence; exact pass count NR	IC tirofiban full-dose bolus; IV infusion for 12 h; ACS antithrombotic therapy	None
**2**	Bulky thrombus in a hyperdominant RCA; primary GEC use		6F Telescope; distal 2.5 × 20-mm balloon-block	Aspiration until a large thrombus was retrieved; exact pass count NR	IC tirofiban full-dose bolus; ACS antithrombotic therapy	None
**3**	Persistent mobile thrombus after 10-min observation following tirofiban		6F Guidezilla II advanced en face to the thrombus	Several suction runs	IC tirofiban full-dose bolus	None
**4**	Three unsuccessful Export aspiration runs; persistent crux thrombus		6F Telescope after selective PD/PL microcatheter therapy	One documented GEC aspiration run	IC tirofiban and heparin; alteplase 2 mg to PL and 1 mg to PD	None
**5**	Persistent subacute stent-edge thrombus; Export failed during index PCI		6F Telescope during repeat procedure	One aspiration sequence; exact pass count NR	IC tirofiban at index PCI; IV infusion for 24 h	OCT before GEC aspiration
**6**	Distal thrombus shift after RCA DES implantation		6F Telescope advanced to distal stent-edge thrombus	One aspiration sequence; exact pass count NR	Half-dose IV tirofiban infusion for 18 h	None
(C)						
**Case**	**Final TIMI Flow**		**Immediate in-Hospital Outcome**			
**1**	3		Uneventful; no residual dissection			
**2**	3		Uneventful; successful discharge			
**3**	3		Uneventful; no stent required; embolic-source work-up negative			
**4**	3		Uneventful; no dissection			
**5**	3		Uneventful			
**6**	3		No immediate adverse event reported; staged LM/LAD PCI completed successfully			

Notes: ACS = acute coronary syndrome; DES = drug-eluting stent; GEC = guide-extension catheter; IC = intracoronary; IV = intravenous; LAD = left anterior descending artery; LM = left main; NR = not reported in the available procedural record; OCT = optical coherence tomography; PD = posterior descending; PL = posterolateral; RCA = right coronary artery; STEMI = ST-elevation myocardial infarction. Exact aspiration-pass counts were not prospectively recorded in several cases.

**Table 2:** Inner diameters and key features of common guide-extension catheters.

GEC Type	Manufacturer	6F Inner Diameter, in (mm)	7F Inner Diameter, in (mm)	8F Inner Diameter, in (mm)	Minimum Guide-Catheter Inner Diameter by Size, in (mm)	Key Design Features and Clinical Relevance
**GuideLiner V3**	Teleflex, Minneapolis, MN, USA	0.056 (1.42)	0.062 (1.57)	0.071 (1.80)	6F: ≥0.070 (1.78) 7F: ≥0.078 (1.98) 8F: ≥0.088 (2.24)	25-cm rapid-exchange segment; half-pipe transition intended to reduce collar-device interaction; 150-cm working length; widely used baseline choice for complex delivery support.
**TrapLiner**	Teleflex, Minneapolis, MN, USA	0.056 (1.42)	0.062 (1.57)	0.071 (1.80)	6F: ≥0.070 (1.78) 7F: ≥0.078 (1.98) 8F: ≥0.088 (2.24)	13-cm rapid-exchange segment; integrated wire-trapping balloon to facilitate catheter exchange while maintaining wire position; hydrophilic coating; combines guide extension and trapping functions.
**Guidezilla II**	Boston Scientific, Maple Grove, MN, USA	0.057 (1.45)	0.063 (1.60)	0.072 (1.83)	6F: ≥0.070 7F: ≥0.078 8F: ≥0.088	Commonly available with a 25-cm guide segment (a long 6F variant also exists); slightly larger lumen than some competitors; platinum-iridium helical collar and hydrophilic coating emphasize visibility and smooth interaction.
**Telescope**	Medtronic, Galway, Ireland	0.056 (1.42)	0.062 (1.57)	-	6F: ≥0.070 7F: ≥0.078	Rapid-exchange segment not specified in the source comparison sheet; SmoothPass concept with tapered distal pushwire and polymer on-ramp/entry port intended to improve device entry; hydrophilic-coated jacket.
**LiquID**	Seigla Medical, Buffalo, MN, USA	0.061 (1.55)	0.071 (1.80)	-	6F: compatible with ≥6F guide catheter (device OD 0.068 [1.73]) 7F: compatible with ≥7F guide catheter (device OD 0.078 [1.98])	15-cm single-lumen distal tube on a 150-cm device; relatively large effective lumen compared with many 6F/7F GECs; coil-reinforced distal segment for kink resistance and radiopacity; silicone coating for lubricity; proximal positioning markers at 95 and 105 cm; color-coded handle. Potentially relevant when larger lumen/support is desirable, but thrombus aspiration remains off-label and lumen size alone does not prove clinical superiority.

**Notes:** Inner diameter is not the sole determinant of deliverability; collar or transition geometry is often the point of stent hang-up or stripping. Deep intubation can improve backup support but may increase the risk of ostial trauma or ischemia; monitor for pressure damping and maintain coaxial alignment. Compatibility with the guide-catheter inner diameter, including 6F thin-wall variants, should be verified before attempting bulky device delivery. LiquID 061/071 dimensions are manufacturer/IFU specifications; the IFU lists compatible guide-catheter sizes rather than a separate minimum guide-catheter inner-diameter threshold.

**Table 3:** Adjunct pharmacology for slow-flow/no-reflow and high-thrombus-burden interventional scenarios.

Therapeutic Category	Drug	Typical Dose	Most Common Adverse Effects
Intracoronary vasodilator	Adenosine	50–200 micrograms IC; common bolus dosing: 60–120 micrograms in the RCA and 120–240 micrograms in the LCA	Atrioventricular block, bradycardia, hypotension, bronchospasm
Intracoronary vasodilator	Diltiazem	400 micrograms IC	Atrioventricular block, hypotension
Intracoronary vasodilator	Nitroprusside	50–200 micrograms IC	Hypotension
Intracoronary vasodilator	Nicardipine	100–200 micrograms IC	Atrioventricular block, hypotension
Intracoronary vasodilator	Nitroglycerin	100–200 micrograms IC	Hypotension
Intracoronary vasodilator	Verapamil	100–250 micrograms IC bolus, administered slowly over 20–30 s	Atrioventricular block, bradycardia, hypotension
GP IIb/IIIa inhibitor	Eptifibatide	180 micrograms/kg bolus via the guiding catheter	Bleeding, thrombocytopenia
GP IIb/IIIa inhibitor	Tirofiban	25 micrograms/kg bolus via the guiding catheter	Bleeding, thrombocytopenia
Other	Epinephrine	100–200 micrograms (maximum 400 micrograms), titrated to effect; 1 mg in 10 mL saline = 100 micrograms/mL; may be administered via the guiding catheter or distally via microcatheter	Tachyarrhythmias, hypertension
Other	Alteplase (rt-PA)	2–5 mg slow bolus via the guiding catheter or distally via microcatheter	Bleeding, distal embolization
Other	Cangrelor	Intravenous dosing: 30 micrograms/kg bolus followed by 4 micrograms/kg/min infusion for 2 h or for the required PCI duration	Bleeding; dyspnea—usually transient and mild to moderate

Notes: Doses are illustrative reference ranges only and do not replace institutional protocols, prescribing information, contraindication screening, or guideline-based pharmacologic management. Final agent selection, route, and dose should reflect vessel size, hemodynamics, rhythm, renal function, bleeding risk, concomitant antithrombotic therapy, and local practice. Intracoronary alteplase and epinephrine are off-label in this context and require highly selective use.

**Table 4:** Potential pitfalls and complications of GEC-assisted thrombus aspiration, with preventive and management measures.

Complication or Pitfall	Prevention and Management
**Ischemic stroke,** ** systemic embolization, or ** ** coronary embolization**	Maintain uninterrupted negative pressure throughout thrombus engagement and withdrawal. If the tip corks, retract the GEC into the guide and remove the GEC ± guide as a closed unit outside the body. Do not release suction while thrombus is engaged. Before further angiography or device delivery, aspirate/back-bleed the guide and confirm free blood return.
**Coronary dissection or ischemia** ** from deep GEC seating**	Advance gently and maintain coaxial alignment. When appropriate, use balloon-anchor or inchworm techniques without force. Stop for resistance, pressure damping, ischemia, or loss of coaxiality; avoid prolonged deep intubation in small, ostial, tortuous, or heavily diseased segments.
**Stent stripping or deformation at the** ** guide-extension collar**	If resistance occurs during stent delivery, avoid forceful advancement. Withdraw carefully and consider guide-extension removal, rotation or coaxial realignment, or gentle balloon flaring of the proximal collar before reattempting delivery. When feasible, prefer low-profile stent platforms when guide extensions are used.
**Air embolism**	Prime and meticulously de-air the GEC, tubing, stopcock, and guide before every pass. Keep the stopcock closed during disconnection and do not inject contrast through a system that may contain air or thrombotic debris.
**Hemodynamic deterioration**	Be prepared to use vasoactive agents and, when needed, mechanical circulatory support in high-risk patients, including those with heart failure or cardiogenic shock.
**Device entrapment or kinking**	Avoid sharp vessel bends, preserve a smooth catheter course, and minimize torquing of the guide-extension catheter.
**Loss of suction or catheter-tip corking**	Stop forward manipulation. Maintain locked suction and withdraw under continuous negative pressure; remove the GEC and, if needed, the guide as a closed unit. Repeated ineffective passes should trigger reassessment or a different strategy.

**Table 5:** Summary of contemporary guideline positions relevant to coronary thrombus aspiration.

Guideline	Routine Manual Aspiration	Selective/Bail-Out Position	Interpretation for GEC-Assisted Aspiration
2023 ESC ACS guidelines [10]	IIIA—Not recommended	May be considered when large residual thrombus persists after opening the vessel with a guidewire or balloon	No validation of GEC use; any application remains selective, off-label, and anatomy-dependent
2025 ACC/AHA/ACEP/NAEMSP/SCAI ACS guidelines [11]	Class 3 (No Benefit) for routine manual aspiration in STEMI undergoing primary PCI	No recommendation establishing routine use of a GEC-based aspiration strategy	The present workflow must not be interpreted as guideline-supported or as an alternative to purpose-built systems

Notes: ACS = acute coronary syndrome; GEC = guide-extension catheter; PCI = percutaneous coronary intervention; STEMI = ST-elevation myocardial infarction. The final column is the authors’ conservative interpretation of guideline implications and is not itself a recommendation.

## Data Availability

No new data were created or analyzed in this study. Data sharing is not applicable to this article.

## Supplementary Materials

The following supporting information can be downloaded at https://www.mdpi.com/article/10.3390/jcm15145582/s1.
