# Supplementary material for: Guide-Extension Catheter-Assisted Bail-Out Thrombus Aspiration During PCI for Thrombus-Rich Acute Coronary Syndromes: Contemporary Review and Clinical Case Examples
Source: J Clin Med. 2026 Jul 16;15(14):5582. doi: 10.3390/jcm15145582 (PMC13413003; doi:10.3390/jcm15145582)
Supplement: Supplementary file 1 [file jcm-15-05582-s001.zip › Supplementary File S1.pdf]

Supplementary File S1

CLINICAL CASES FROM REAL-WORLD PRACTICE

Clinical case #1

A 48-year-old Caucasian male smoker (30-pack-years) with arterial hypertension and no other known comorbidities was transferred to our center with emergent helicopter transport due to chest pain and subacute inferoposterior STEMI with symptom to door time of about 12 hours. The patient was previously loaded in the emergency services with 60 mg of prasugrel, 300 mg of ASA and 5,000 units of intravenous unfractionated heparin. The immediate coronary angiography with 6F JR4 catheter initially showed occluded right coronary artery (RCA) in the proximal segment (**Supplementary Figure S1**). Due to the high thrombotic burden, a 6F Guidezilla II guide extension catheter (Boston Scientific) was advanced over the wire with 2.5 x 15 mm distal SC balloon anchored in distality of the vessel inflated at nominal pressure - distal balloon *"block"* technique (middle and right upper panel of Supplementary Figure 1). The full dose of intracoronary tirofiban adjusted to body mass was administered (58 mL bolus). The rationale behind the SC balloon use in this setting was to position it distally with respect to thrombotic lesion as a sort of a *"filtering"* device to prevent potential distal embolization of thrombus debris. The Guidezilla II GEC was deeply intubated into the vessel, but no significant pressure dumping occurred. Thrombotic mass was then negotiated with the tip of the GEC *"en face"* under negative pressure from *"top to bottom"* and then it was slowly retracted with *"bottom to top"* motion. A large quantity of thrombus material was retrieved through the GEC and documented on the procedure table (Supplementary Figure 1, lower left panel). Then, the whole GEC system was removed from the patient body under negative pressure and was thoroughly inspected and flushed outside of the patient body. Since patient was hemodynamically stable after these measures, without chest pain and ECG changes and last cine documented improvement of result we also decided to take the wire out and introduce a new 6F Amplatz AL1 guide catheter to achieve better support for PCI finalization. RCA was then selectively intubated again and the first contrast injection following thrombus aspiration was performed (Supplementary Figure 1, middle lower panel). After multiple balloon dilatations, a long 3.0 x 40 mm sirolimus-eluting stent was implanted with balloon inflated @ 20 atm. Finally, a TIMI-3 flow was established through large and dominant RCA with no residual dissection (Supplementary Figure 1, right lower panel). The continuous intravenous infusion of tirofiban (21 mL/h) for 12 hours was continued in the Coronary Care Unit. The post-interventional course was uneventful.

Clinical case #2

A 48-year-old Caucasian male patient with history of smoking has presented with inferior STEMI. The angiography showed hyper dominant RCA with 100% proximal occlusion, after wire pass shown to be tamponed with thrombotic masses (**Supplementary Figure S2**, upper three panels). RCA was engaged with 6F Amplatz AL1 catheter and Fielder FC wire with Corsair Pro microcatheter was used to cross the lesion and then the exchange for the workhorse wire was performed. Intracoronary bolus of tirofiban adjusted for body mass (67 mL) was given to patient after which we used anchoring distal balloon *"block"* technique with semi-compliant 2.5 x 20 mm balloon inflated to nominal pressure to prevent distal embolization while 6F Telescope GEC was used to evacuate a large quantity of red thrombus mass (Supplementary Figure 2, lower left and middle panel). Finally, two drug-eluting stents were implanted (5.0 x 26 mm proximally and 3.0 x 33 mm distally). TIMI-3 flow was finally restored within the vessel (Supplementary Figure 2, lower right panel) with uneventful further clinical course and successful patient discharge.

Clinical case #3

A 47-year-old Caucasian male patient with a history of smoking and arterial hypertension presented with an inferior STEMI. Diagnostic coronary angiography revealed a hazy and floating oval-shaped thrombotic mass in the middle segment of the RCA that obstructed the flow for 80% while the full periphery of the vessel could be visualized (**Supplementary Figure S3**, upper left panel). RCA was selectively intubated with JR4 guide catheter and wired with a workhorse wire and intracoronary tirofiban adjusted for body mass (45 mL bolus) was administered. We then observed a patient for additional 10 minutes to see if the thrombus will dissolve. However, control angiography revealed no resolution of the mass although it had a slightly different angiographic appearance. We then delivered a 6F Guidezilla II GEC "en face" against the mass and performed several suction runs under negative pressure (Supplementary Figure 3, lower left panel) after we managed to evacuate a large solitary well-consolidated thrombotic mass on the table (Supplementary Figure 3, lower right panel). Final angiography documented patent TIMI-3 flow, no angiographic signs of stenosis with full visualization of vessel periphery and no dissection (Supplementary Figure 3, upper right panel). We finished the case without the need for stenting or drug-coated balloon application. Subsequent intrahospital diagnostic work-up for potential embolization sources/patent foramen ovale was negative. Further clinical course was uneventful, and patient was discharged home on short-duration DAPT.

Clinical case #4

A 30-year-old male patient of East Asian descent with no known comorbidities presented with inferolateral STEMI. The vessel was intubated with 6F MP guide catheter while the first contrast injection showed 100% thrombotic occlusion of mid RCA after which the lesion was crossed and posterolateral branch was wired with a workhorse wire (**Supplementary Figure S4**, upper panels). Slight reperfusion showed that the vessel was dominant and gave off PD and PL branches. Intracoronary tirofiban and heparin, adjusted for body mass, were administered. Large thrombotic masses were documented at the vessel crux, protruding and obstructing flow in both the PD and PL branch after which additional wire was used to wire PD branch. Following this, a 6F Export thrombus aspiration catheter was used and three aspiration runs did not achieve thrombus suction and provided inadequate result. Since no significant improvement of flow was achieved, we used FineCross MG microcatheter to engage PD and PL branches selectively. We used a small dose of intracoronary alteplase that was injected into PL branch (2 mg) and PD branch (1 mg) after which we performed another aspiration run with 6F Telescope GEC that evacuated a significant quantity of thrombotic material (Supplementary Figure 4, lower left panel). A significant improvement of flow was achieved after which we performed plain old balloon angioplasty (POBA) of both PD and PL branches with non-compliant 2.0 x 20 mm balloons at high-pressure inflations. We established a brisk TIMI-3 flow through RCA with no dissection and full visualization of periphery (Supplementary Figure 4, lower right panel). Further clinical course was uneventful.

Clinical case #5 - adjacent technical example: subacute stent-edge thrombus after recent PCI

A 55-year-old Caucasian man with arterial hypertension was admitted due to the chest pain in the setting of chronic coronary syndrome and during the index coronary angiography a sub occlusive 99% thrombotic stenosis of the proximal LAD was revealed. Patient was stented with one 3.5 x 33 mm DES, and the stent was optimized via optical coherence tomography (OCT) guidance with 4.0 x 12 mm and 4.5 x 15 mm non-compliant balloons, however, a residual thrombotic mass in the proximal LAD persisted after these actions. The operator tried to make manual aspiration with dedicated Export 6F catheter, however, without success. Finally, operator decided to administer intracoronary tirofiban in full bolus dose (46 mL) after which a partial resolution of thrombus was achieved and the intravenous infusion of tirofiban was continued for 24 hours in the Coronary Care Unit. Since there were no ECG changes, no chest pain reported and patient had normal vital signs, procedure was completed. During this procedure patient was loaded with 300 mg of ASA, 180 mg of ticagrelor and received 10,000 IU of unfractionated heparin. Patient returned 7 days later in the catheterization laboratory with recurring chest

pain, in the clinical context of unstable angina. Angiography revealed a floating thrombus mass in the vicinity of proximal stent edge (**Supplementary Figure S5**, upper left panel), LAD was wired and OCT run was performed (Supplementary Figure 5, upper right panel). The OCT analysis revealed normal expansion and apposition of previously implanted DES, however, a significant intraluminal obstruction with subacute white thrombus was detected in the proximal segment of the stent edge (Supplementary Figure 5, lower left panel). Thrombus aspiration with 6F Telescope GEC was performed evacuating a large white thrombotic mass after which patent TIMI-3 flow was achieved in the LAD (Supplementary Figure 5, lower right panel). The rest of clinical course was uneventful.

Clinical case #6 - adjacent technical example: thrombus shift after stent deployment

A 73-year-old male patient was admitted for inferolateral STEMI. The RCA was hyper dominant and occluded in a high proximal segment. The patient had a significant disease in the left coronary artery circulation but the culprit for the acute event was RCA. The occlusion was passed with the polymer-jacketed workhorse wire, and the segment was predilated with non-compliant 3 x 20 mm balloon followed by one DES implantation (4.0 x 38 mm @ 20 atm) as shown in **Supplementary Figure S6** (upper left panel). However, a thrombus shift occurred after stenting, and a thrombotic mass was revealed caudal to distal stent edge (Supplementary Figure 6, upper mid panel). The 6F Telescope GEC was advanced to the site of the thrombus (Supplementary Figure 6, upper right panel) and was completely removed, as shown in Supplementary Figure 6 (lower left panel). A post-dilation and optimization of the implanted DES was performed with an NC 4.5 x 20 mm balloon. Finally, a TIMI-3 flow was achieved through the vessel (Supplementary Figure 6, lower mid panel) while the aspirated thrombotic mass was revealed on the table (as shown in Supplementary Figure 6, lower right panel). A half-dose continuous intravenous infusion of tirofiban was continued in the Coronary Care Unit in the dose of 7 mL/h for 18 hours. In second intrahospital sitting, the PCI of LM/LAD was planned and eventually successfully performed.

FIGURE LEGENDS

Supplementary Figure S1. Coronary angiogram showing thrombotic occlusion of the right coronary artery (RCA) that was treated with thrombus aspiration using Guidezilla IITM guide extension catheter with SC balloon anchored and inflated distally to prevent further thrombotic embolization - distal "balloon block" technique. A large quantity of red thrombus was extracted that secured further antegrade flow while final PCI was performed with implantation of a long sirolimus drug-eluting stent (3.0 x 40 mm inflated at 20 atm). A final angiogram documented successfully restored TIMI-3 flow with no residual dissection or complications at the vessel periphery.

Supplementary Figure S2. Coronary angiogram showing thrombotic occlusion of the proximal RCA that was tamponed with thrombotic masses. A 6F TelescopeTM GEC was used for thrombus aspiration in the mid segment with distal 2.5 x 20 mm semi-compliant "balloon block" technique employed to prevent distal embolization. A large quantity of red thrombus was evacuated with final PCI performed with two DES implantations (5.0 x 26 mm proximally and 3.0 x 33 mm distally). A TIMI-3 flow with no dissection and no complications at the vessel periphery.

Supplementary Figure S3. Coronary angiogram showed a hazy and oval-shaped solitary thrombotic mass in the mid RCA that obstructed flow for 80%. A 6F GEC was delivered "en face" against the mass that was successfully aspirated after several suction runs. A dominantly red thrombotic mass with some outer white lining was evacuated. Following this aspiration, a brisk TIMI-3 flow was achieved with full visualization of periphery, no dissection and no significant stenoses in the RCA.

Supplementary Figure S4. Coronary angiogram showed a thrombotic occlusion of mid RCA that was engaged with 6F MP catheter. Both PD and PL branches were wired while thrombotic masses were centralized at the crux region and protruded in both branches. Thrombus aspiration with 6F TelescopeTM GEC, facilitated with selective intracoronary application of alteplase in both PD and PL branches were performed. A large red thrombus was evacuated with GEC while PD and PL branches of the RCA was finally treated with plain old balloon angioplasty (NC balloon 2.0 x 20 mm @ 20 atm in both branches). TIMI-3 flow was restored through RCA with uneventful further course.

Supplementary Figure S5. Coronary angiogram showing intraluminal thrombotic mass near the proximal stent edge in the proximal LAD in an unstable-angina presentation 7 days after index PCI. OCT demonstrated significant intraluminal obstruction by subacute white thrombus with otherwise acceptable stent expansion/apposition. Because conventional aspiration was unsuccessful during the index procedure, this case is presented only as an adjacent technical example of GEC-assisted thrombus retrieval rather than a representative primary PCI high-thrombus-burden indication.

Supplementary Figure S6. Blunt proximal RCA occlusion treated with wiring, predilatation, and DES implantation, followed by distal thrombus shift after stent deployment. GEC-assisted aspiration was used to retrieve the shifted thrombus and restore TIMI-3 flow. This case illustrates management of post-stenting thrombus shift and should not be interpreted as a primary refractory-thrombus indication for routine GEC-assisted aspiration.
