# Supplementary figures and images for: Guide-Extension Catheter-Assisted Bail-Out Thrombus Aspiration During PCI for Thrombus-Rich Acute Coronary Syndromes: Contemporary Review and Clinical Case Examples
Source: J Clin Med. 2026 Jul 16;15(14):5582. doi: 10.3390/jcm15145582 (PMC13413003; doi:10.3390/jcm15145582)

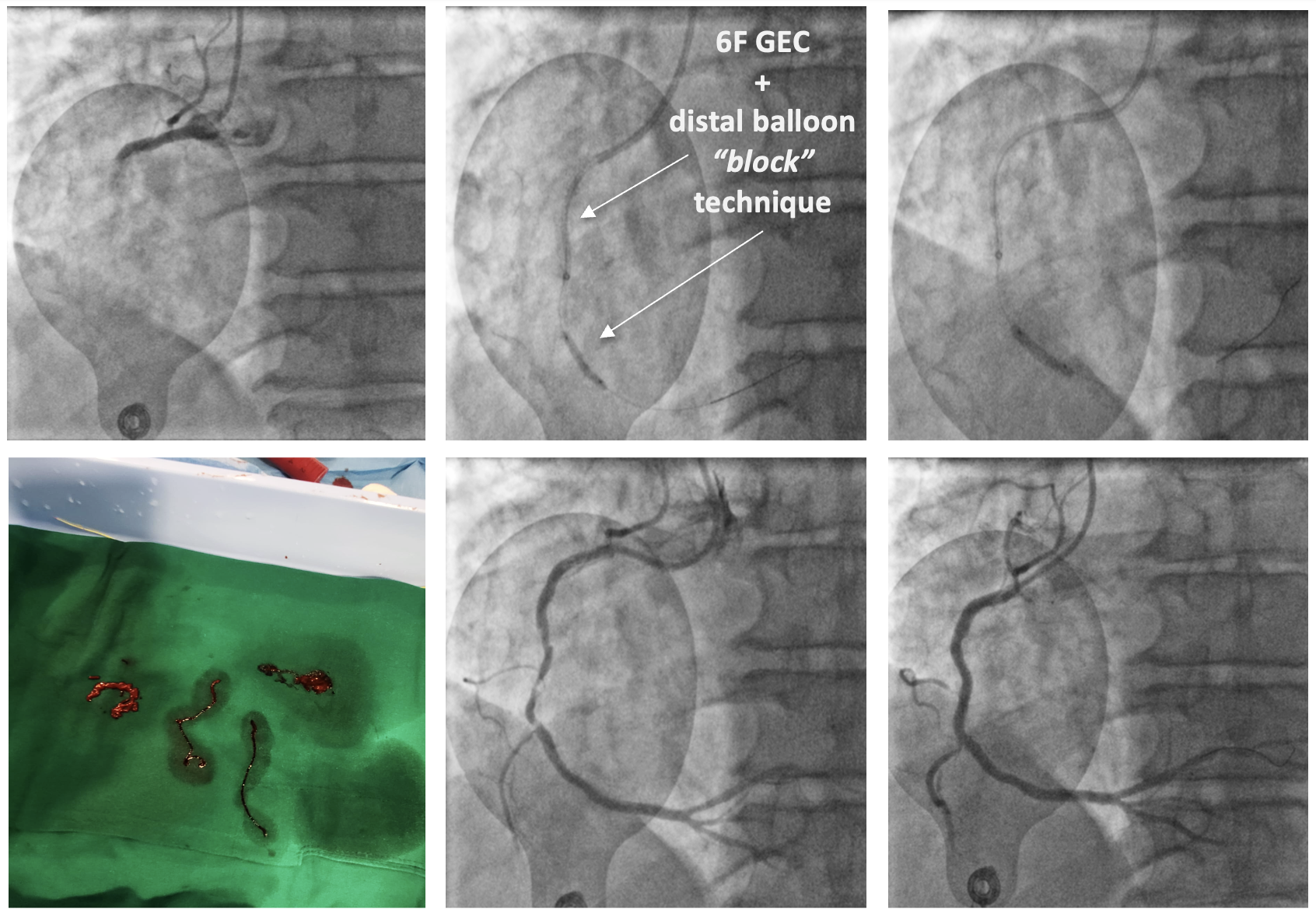

Supplement: Supplementary file 1 [file jcm-15-05582-s001.zip › Supplementary Figure S1.tif]

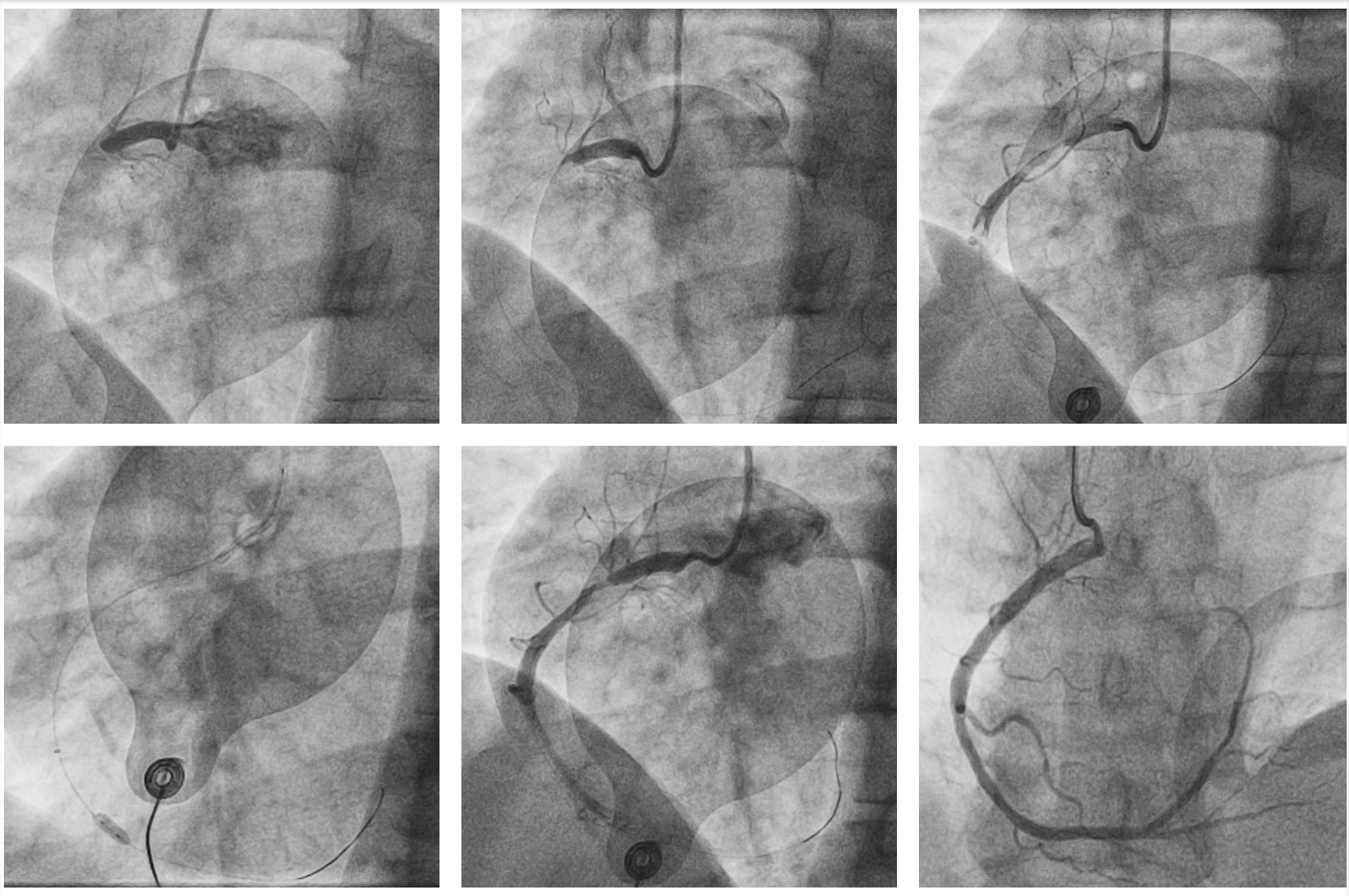

Supplement: Supplementary file 1 [file jcm-15-05582-s001.zip › Supplementary Figure S2.tif]

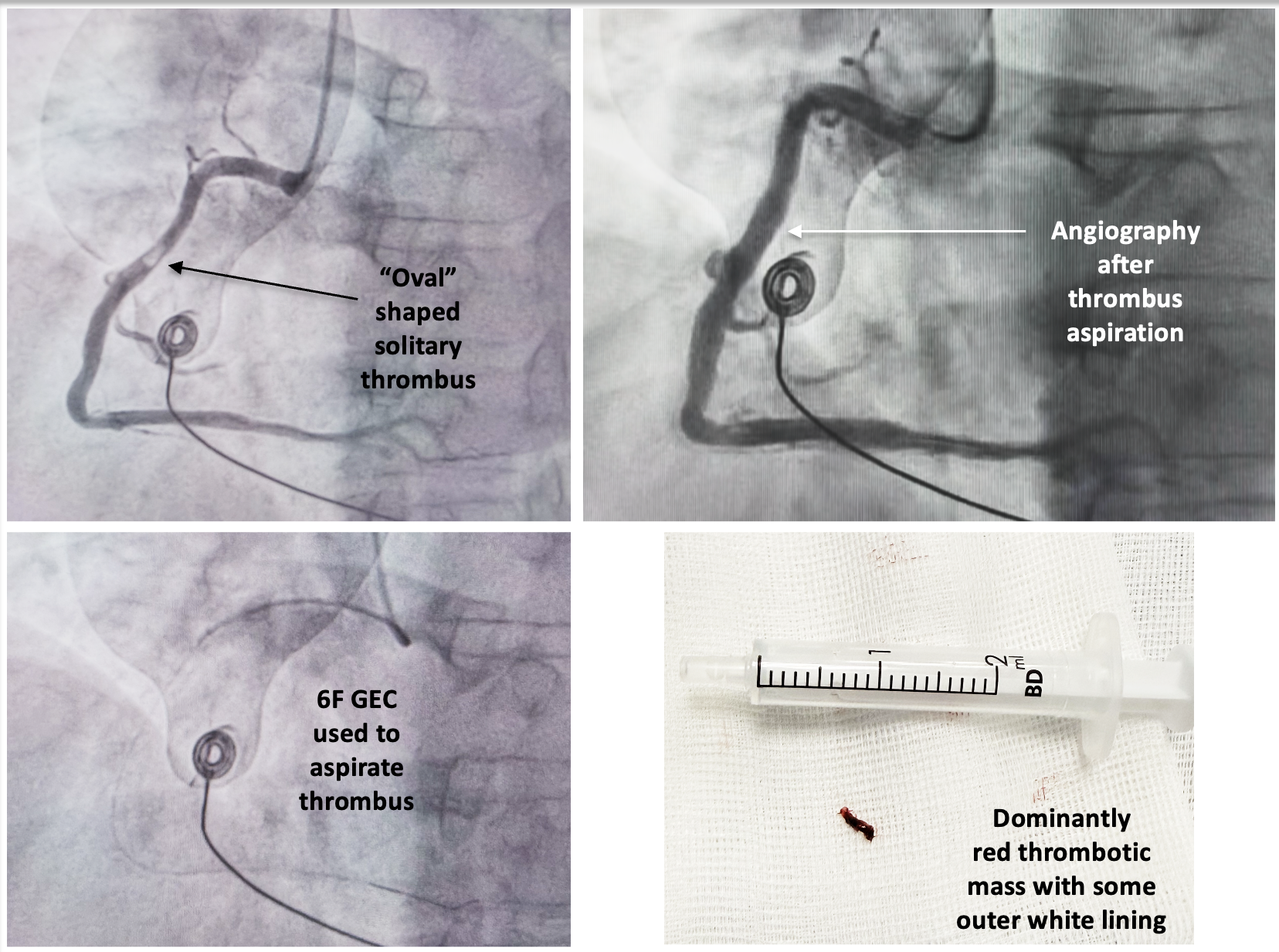

Supplement: Supplementary file 1 [file jcm-15-05582-s001.zip › Supplementary Figure S3.tif]

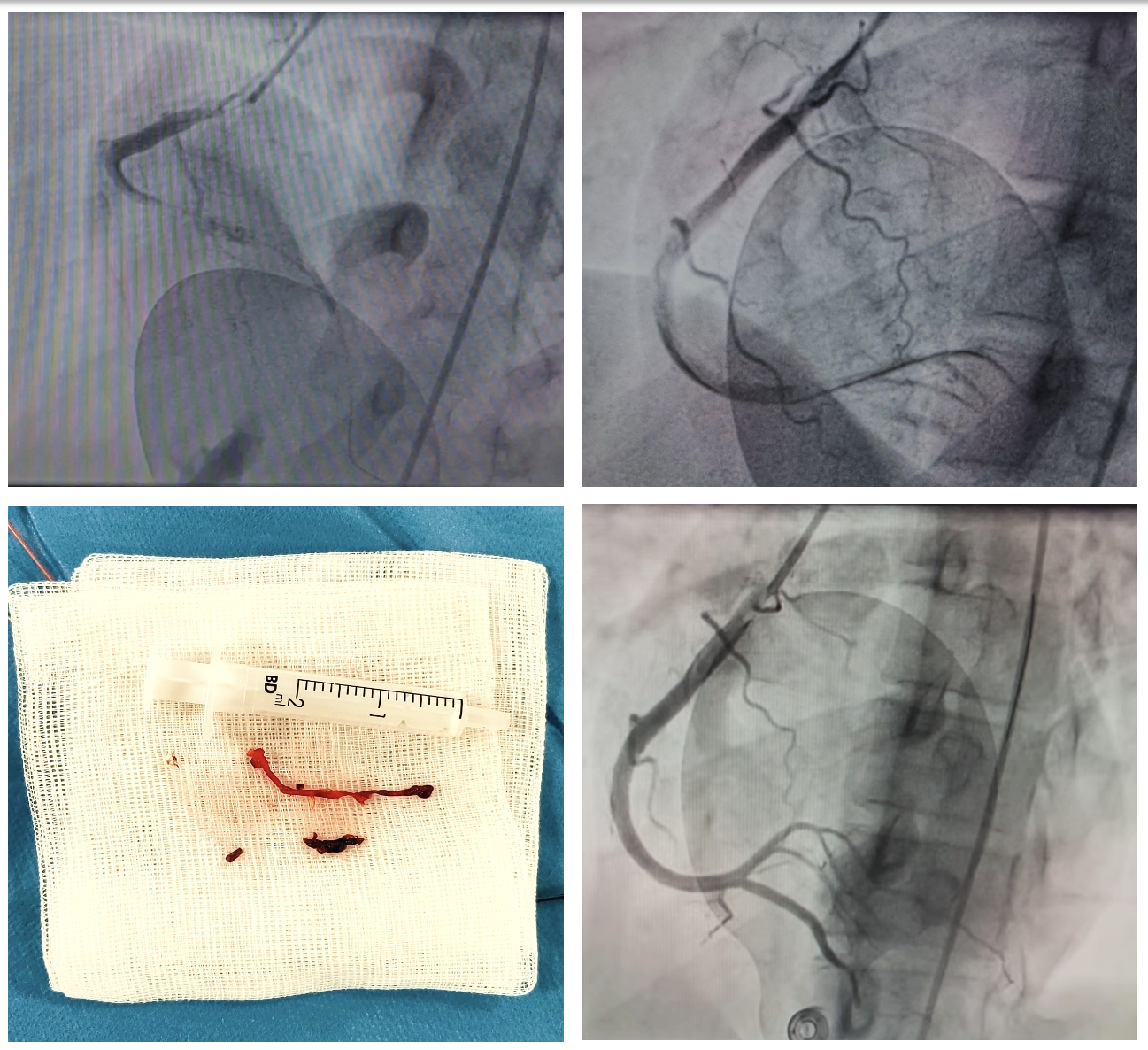

Supplement: Supplementary file 1 [file jcm-15-05582-s001.zip › Supplementary Figure S4.tif]

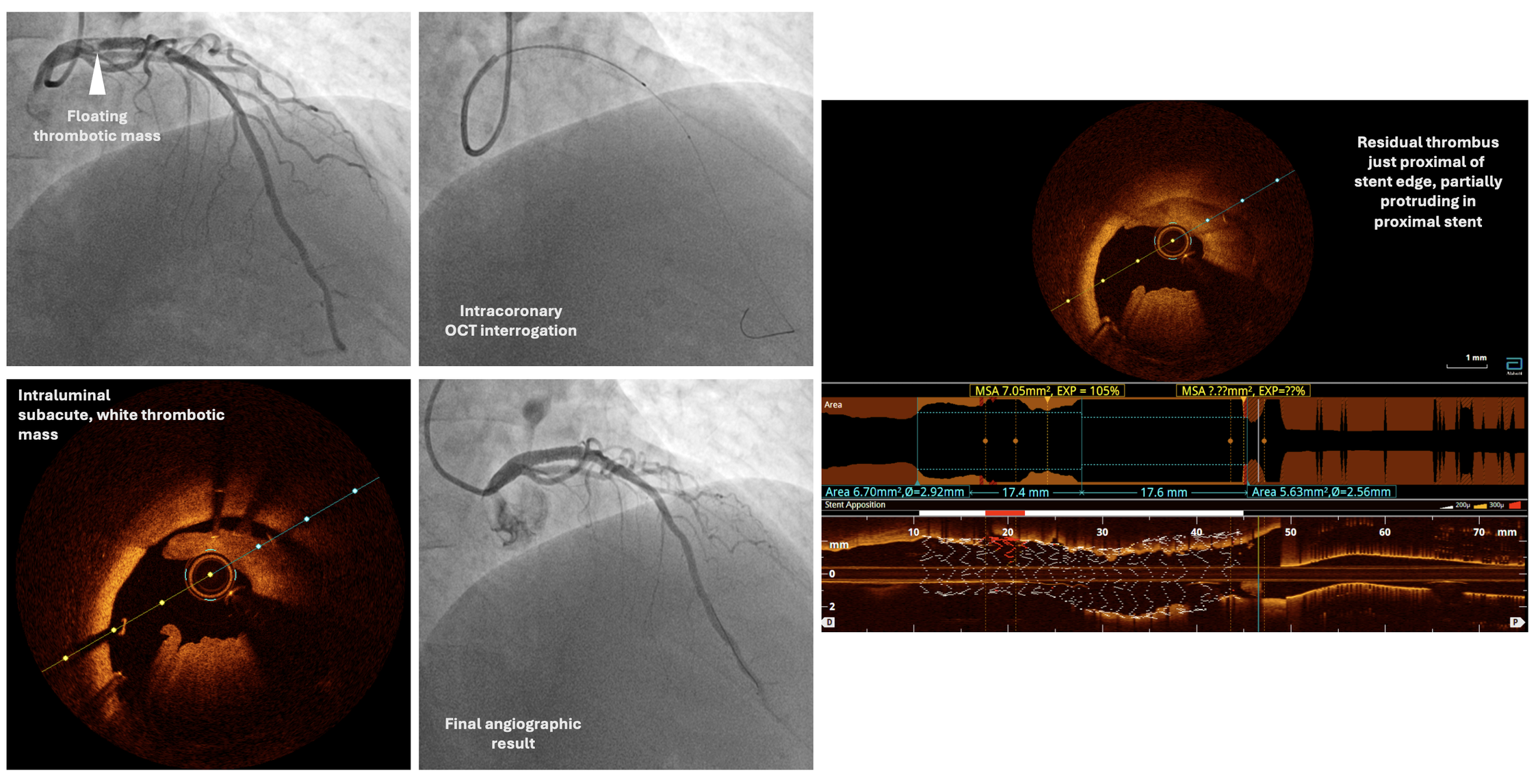

Supplement: Supplementary file 1 [file jcm-15-05582-s001.zip › Supplementary Figure S5.tif]

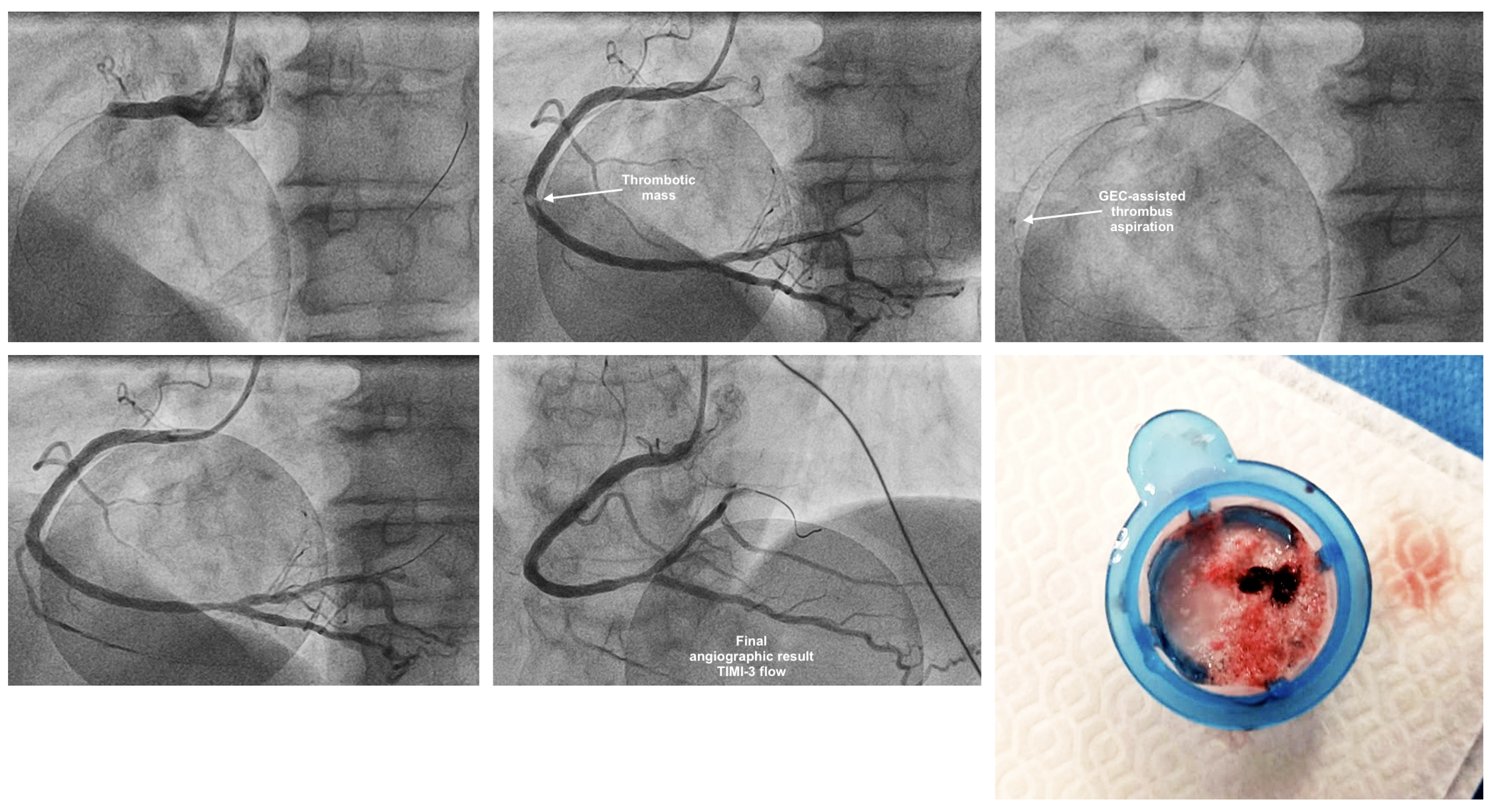

Supplement: Supplementary file 1 [file jcm-15-05582-s001.zip › Supplementary Figure S6.tif]
